# Aberrant Hedgehog Ligands Induce Progressive Pancreatic Fibrosis by Paracrine Activation of Myofibroblasts and Ductular Cells in Transgenic Zebrafish

**DOI:** 10.1371/journal.pone.0027941

**Published:** 2011-12-02

**Authors:** In Hye Jung, Dawoon E. Jung, Young Nyun Park, Si Young Song, Seung Woo Park

**Affiliations:** 1 Postgraduate School of National Core Research Center for Nanomedical Technology, Institute of Gastroenterology, Yonsei University College of Medicine, Seoul, Republic of Korea; 2 Brain Korea 21 Project for Medical Science, Yonsei University College of Medicine, Seoul, Republic of Korea; 3 Department of Pathology, Institute of Gastroenterology, Yonsei University College of Medicine, Seoul, Republic of Korea; 4 Department of Internal Medicine, Institute of Gastroenterology, Yonsei University College of Medicine, Seoul, Republic of Korea; National University of Singapore, Singapore

## Abstract

Hedgehog (Hh) signaling is frequently up-regulated in fibrogenic pancreatic diseases including chronic pancreatitis and pancreatic cancer. Although recent series suggest exclusive paracrine activation of stromal cells by Hh ligands from epithelial components, debates still exist on how Hh signaling works in pathologic conditions. To explore how Hh signaling affects the pancreas, we investigated transgenic phenotypes in zebrafish that over-express either Indian Hh or Sonic Hh along with green fluorescence protein (GFP) to enable real-time observation, or GFP alone as control, at the ptf1a domain. Transgenic embryos and zebrafish were serially followed for transgenic phenotypes, and investigated using quantitative reverse transcription-polymerase chain reaction (qRT-PCR), in situ hybridization, and immunohistochemistry. Over-expression of Ihh or Shh reveals virtually identical phenotypes. Hh induces morphologic changes in a developing pancreas without derangement in acinar differentiation. In older zebrafish, Hh induces progressive pancreatic fibrosis intermingled with proliferating ductular structures, which is accompanied by the destruction of the acinar structures. Both myofibroblasts and ductular are activated and proliferated by paracrine Hh signaling, showing restricted expression of Hh downstream components including Patched1 (Ptc1), Smoothened (Smo), and Gli1/2 in those Hh-responsive cells. Hh ligands induce matrix metalloproteinases (MMPs), especially MMP9 in all Hh-responsive cells, and transform growth factor-ß1 (TGFß1) only in ductular cells. Aberrant Hh over-expression, however, does not induce pancreatic tumors. On treatment with inhibitors, embryonic phenotypes are reversed by either cyclopamine or Hedgehog Primary Inhibitor-4 (HPI-4). Pancreatic fibrosis is only prevented by HPI-4. Our study provides strong evidence of Hh signaling which induces pancreatic fibrosis through paracrine activation of Hh-responsive cells *in vivo*. Induction of MMPs and TGFß1 by Hh signaling expands on the current understanding of how Hh signaling affects fibrosis and tumorigenesis. These transgenic models will be a valuable platform in exploring the mechanism of fibrogenic pancreatic diseases which are induced by Hh signaling activation.

## Introduction

Hh ligands are secreted glycoproteins and they initiate hedgehog signaling upon binding to Patched (Ptc) receptors. The signaling is transmitted through Smoothened (Smo)'s activation, resulting in the Gli-mediated transcriptional up-regulation of Hh target genes. This signaling plays a critical role in both physiologic and pathologic conditions by participating in cell differentiation and tissue patterning during early embryonic development and in tissue homeostasis as well as tumorigenesis in adult organs [1], [2]. The Desert Hedgehog (Dhh) is known to be largely restricted by gonads during embryonic development [3], [4]. On the other hand, the Indian Hedgehog (Ihh) and Sonic Hedgehog (Shh) are expressed in various organs, including the endoderm and the gastrointestinal tract; thereby showing an overlapped expression, suggesting that they are functionally redundant [5], [6].

The pancreas is one of the organs where Hh signaling is strictly controlled. Although inactivation of Hh signaling is a crucial event for proper pancreatic development and differentiation, this signaling is frequently reactivated in fibrogenic pancreatic diseases. For instance, chronic pancreatitis and pancreatic ductal adenocarcinoma, with several components of Hh pathway are frequently and often markedly up-regulated in early stages of those conditions [7], [8], [9].Thus, these are representative of pancreatic diseases accompanying prominent desmoplastic reaction, in which active Hh signaling is somehow involved in fibrogenesis. An *in vitro* study revealed enhanced migration of pancreatic stellate cells by exogenous Ihh [10]. Moreover, the impact of Hh signaling on fibrosis does not seem to be confined to the pancreas. It also exerts an effect on fibrosis of the lungs, bile duct, and liver. This suggests that a similar paradigm works in various organs [11], [12], [13].

It has been well-documented that Hh signaling relies on paracrine action for proper patterning of the gastrointestinal tract during murine development [14]. Though evidence from recent observation has suggested a paracrine mechanism for Hh signaling in both physiologic and pathologic conditions [15], an autocrine mechanism cannot be completely excluded in certain types of malignancy [16], [17]. These findings reflect the possible existence of cell-type or organ-dependency, necessitating further clarification of Hh signaling. This raises a question regarding pathologic consequences of aberrantly expressed Hh ligands in the exocrine pancreas.

Since the early 1980s, the zebrafish has been widely used for the study of genetics and developmental biology, and is often exploited as a disease model [18]. Conservation of the genetic program strengthens the power of using the zebrafish model in simulating human diseases. Frequently, the orthologs of the human gene are duplicated in zebrafish. The orthologs of Ihh and Shh are also duplicated in zebrafish, suggesting the existence of redundancy within subtypes. Recent advances in technology have facilitated the establishment of transgenic zebrafish with greater efficiency and convenience. The implication of Hh signaling and pancreatic fibrosis has been firmly documented as a result of *in vitro* studies [10], specimens of diseased pancreas [19], and xenograft model of pancreatic cancer [20]. Nonetheless the direct effect of an aberrant Hh expression on the pancreas has not clearly established. In an earlier study [21], the authors demonstrated that precancerous lesions developed in the pancreas of Pdx1-Shh transgenic mice. However, they did not mention any findings which are relevant to pancreatic fibrosis. Therefore, in this paper, we have generated transgenic zebrafish in which Ihha or Shha is over-expressed in the ptf1a domain to investigate the effects of Hh ligands in the exocrine pancreas. The results show *in vivo* evidence that Hh ligands cause pancreatic fibrosis by paracrine activation of myofibroblasts, as well as ductular cells.

## Results

### Targeted expression of transgenes and short-term phenotypes

**Figure 1:**
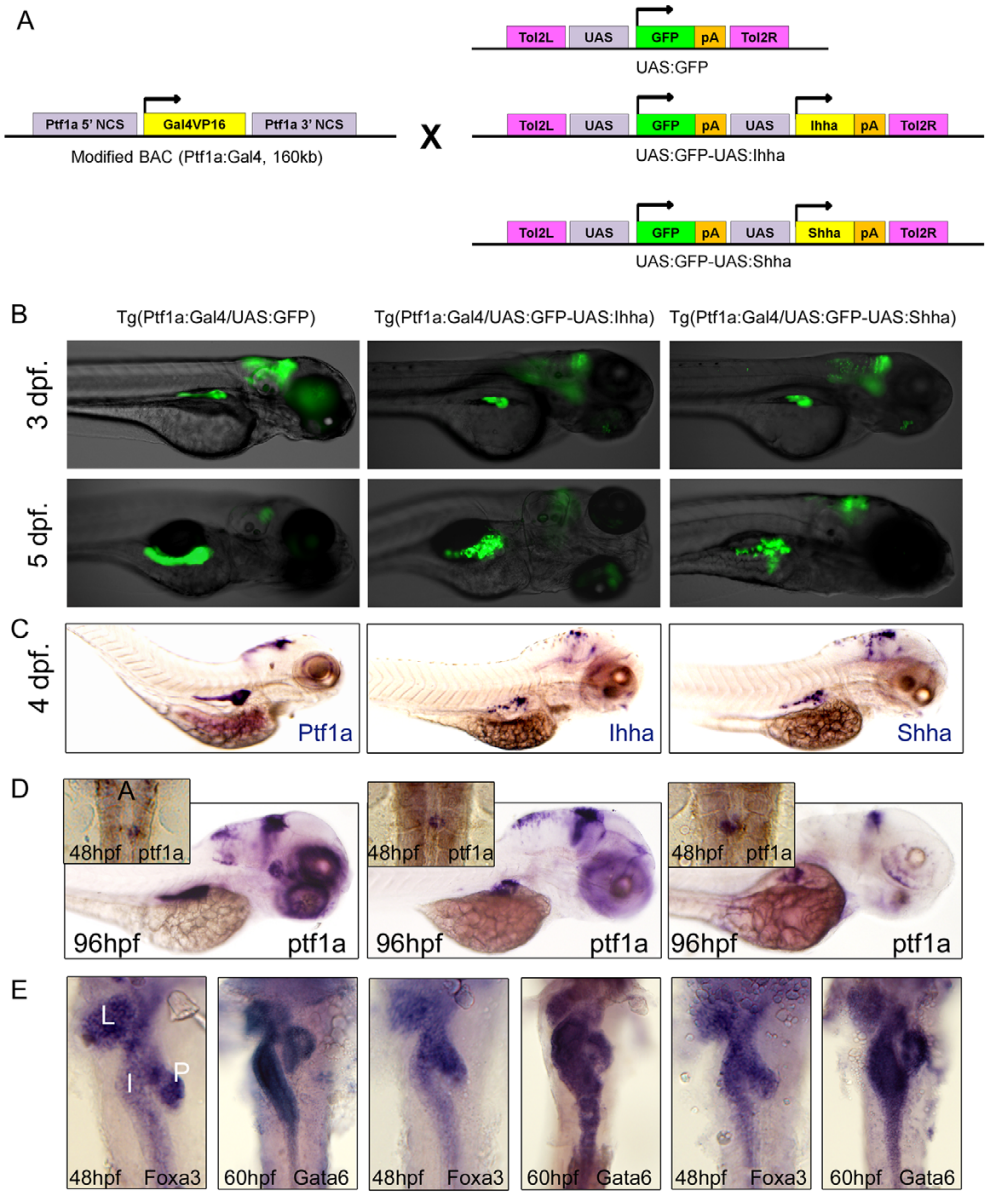
Short-term phenotypes. (**A**) Transgenesis strategy. (**B, C**) Inverted fluorescence and transgene ISH images show mosaic pattern of transgene expression in Hh ligand-expressing embryos. (**D**) Whole mount ISH for ptf1a at 48 and 96 hpf. Inlet figures are dorsal views with anterior to the top. A, anterior. Hh over-expression did not impair migration of ptf1a-expressing exocrine progenitor cells, showing ptf1a positive exocrine cells surrounding principal islet at 48 hpf. (**E**) Whole mount ISH for Foxa3 and Gata6, endodermal markers during development. Dorsal views with anterior to the top. The FoxA3 and Gata6 are properly expressed in the liver, intestine, and exocrine pancreas, and the endodermal morphologies are not affected by Hh over-expression. L, Liver; I, Intestine; P, Exocrine pancreas.

In order to express transgenes from a zebrafish pancreas, we took advantage of Tg(Ptf1a:Gal4) zebrafish [22] which had previously been established by bacterial artificial chromosome (BAC) and allowed binary expression by Gal4-UAS system. Transgene constructs were generated to co-express either Ihha or Shha along with green fluorescence protein (GFP) which enabled real-time observation (Fig. 1A). From each construct, 7 independent transgenic lines were successfully established: Tg(Ptf1a-Gal4/UAS:GFP-UAS:Ihha), Tg(Ptf1a-Gal4/UAS:GFP-UAS:Shha), and Tg(Ptf1a-Gal4/UAS:GFP). The transgene expression levels estimated by GFP, however, varied among the F1 progenies depending on their parental zebrafish. All independent lines were separately maintained.

When transgene expression was evaluated by GFP expression or by ISH, it was found to be spatiotemporally restricted to the Ptf1a domain (Fig. 1B, C). In control embryos, GFP was expressed throughout the whole exocrine pancreas. In Hh ligand-expressing embryos, patterns of transgene expression were not homogeneous throughout the whole exocrine pancreas, but rather, were mosaic for GFP and Hh ligands expression somewhat due to an unknown cause. Acinar cells surrounding the principal islet tended to show more robust expression of the transgenes. In developing zebrafish, the ptf1a-positive cells first appear at the left side of the endoderm, migrate across the midline, and eventually encircle the principal islet at 48 hpf. The migration of the exocrine progenitor cells was not affected by Hh expression, showing the doughnut-shaped ptf1-expressing exocrine pancreas at 48 hpf (Fig. 1D, 2A). Next, in order to visualize the developing endoderm, ISH was performed for endodermal markers, FoxA3and Gata6 at 48 and 60 hpf, respectively [23], [24].These transcriptional factors were properly induced in the liver, intestine, and exocrine pancreas. Also, endodermal morphologies were not deranged by Hh over-expression (Fig. 1E).

**Figure 2:**
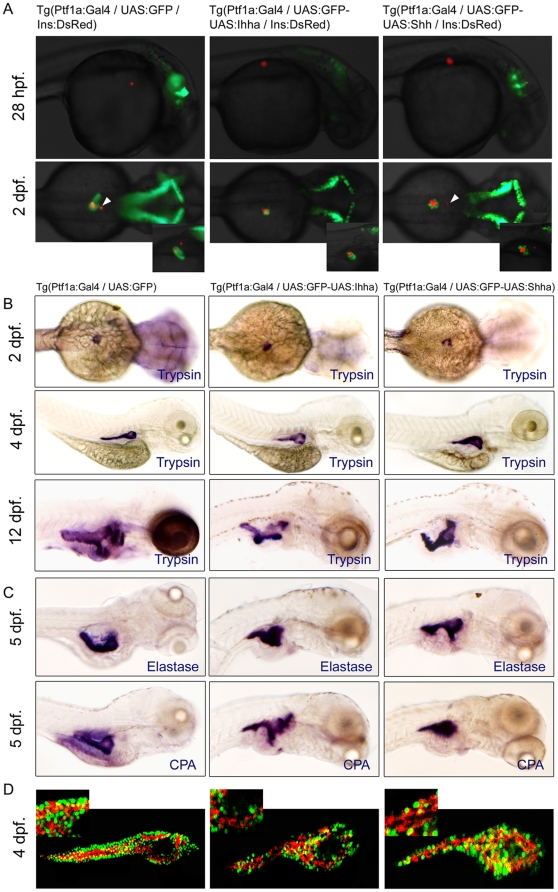
Unaffected endocrine and exocrine differentiation by Hh over-expression. (**A**) Fluorescence images showing the endocrine (RFP) and exocrine pancreas (GFP). When each transgenic fish is crossed with Ins-DsRed zebrafish, the development of insulin-expressing endocrine pancreas is not impaired by Hh over-expression. A smaller dot-like insulin-positive structure (white arrowheads) which corresponds to the anterior endocrine cells is observed in approximately half of the control and Hh-expressing embryos. (**B, C**) Whole mount ISH for trypsin, elastase, and carboxypeptidase A (CPA) at different time points. Over-expression of Hh ligands does not compromise the exocrine differentiation of the zebrafish pancreas, as evidenced by the proper and timely expression of trypsin. Expression of the other exocrine markers is also unaffected by Hh over-expression. Hh over-expression, however, induces subtle morphologic changes of the exocrine pancreas, showing a short, slender, and tortuous posterior pancreas compared to those of controls, which is evident by ISH for exocrine markers at 4 and 5 dpf and exaggerated at 12 dpf. (**D**) Confocal images of immunofluorescence staining for CPA. Regardless of transgene (GFP) expression, most acinar cells express CPA, suggesting unaffected exocrine differentiation by Hh over-expression. Lat., lateral.

The endocrine or exocrine differentiation was not compromised by Hh over-expression (Fig. 2). To visualize the endocrine pancreas, each line was crossed with Ins-DsRed transgenic zebrafish. The emergence of RFP-positive endocrince cells was not different from that of control (Fig. 2A). The anterior endocrine cells appear as a small dot like structure at the rostral side of the principal islet and are visible in approximately 50% of the control embryos, which was also not affected by Hh over-expression (Fig. 2A). The exocrine differentiation was evaluated by trypsin, elastase, and carboxypeptidase A (CPA) expression. The appearance of trypsin expression at 48 hpf did not temporally differ from that of the control embryos. The expression of other exocrine markers, such as elastase and CPA were also properly induced (Fig. 2B, C). Aberrant Hh expression, however, caused morphologic changes of exocrine pancreas when estimated by GFP expression or by ISH. The exocrine pancreas in Hh-expressing embryos showed a short, slender, and tortuous posteriorly-growing pancreas with a relatively prominent head compared to that of the control, which was evident at 4 and 5 dpf and exaggerated at 12 dpf (Fig. 2B, C). Confocal imaging of CPA immunofluorescence staining revealed proper exocrine differentiation of individual acinar cells regardless of transgene expression, suggesting that the exocrine differentiation program was not affected by Hh over-expression.

### Aberrant Hedgehog ligands cause pancreatic fibrosis

**Figure 3:**
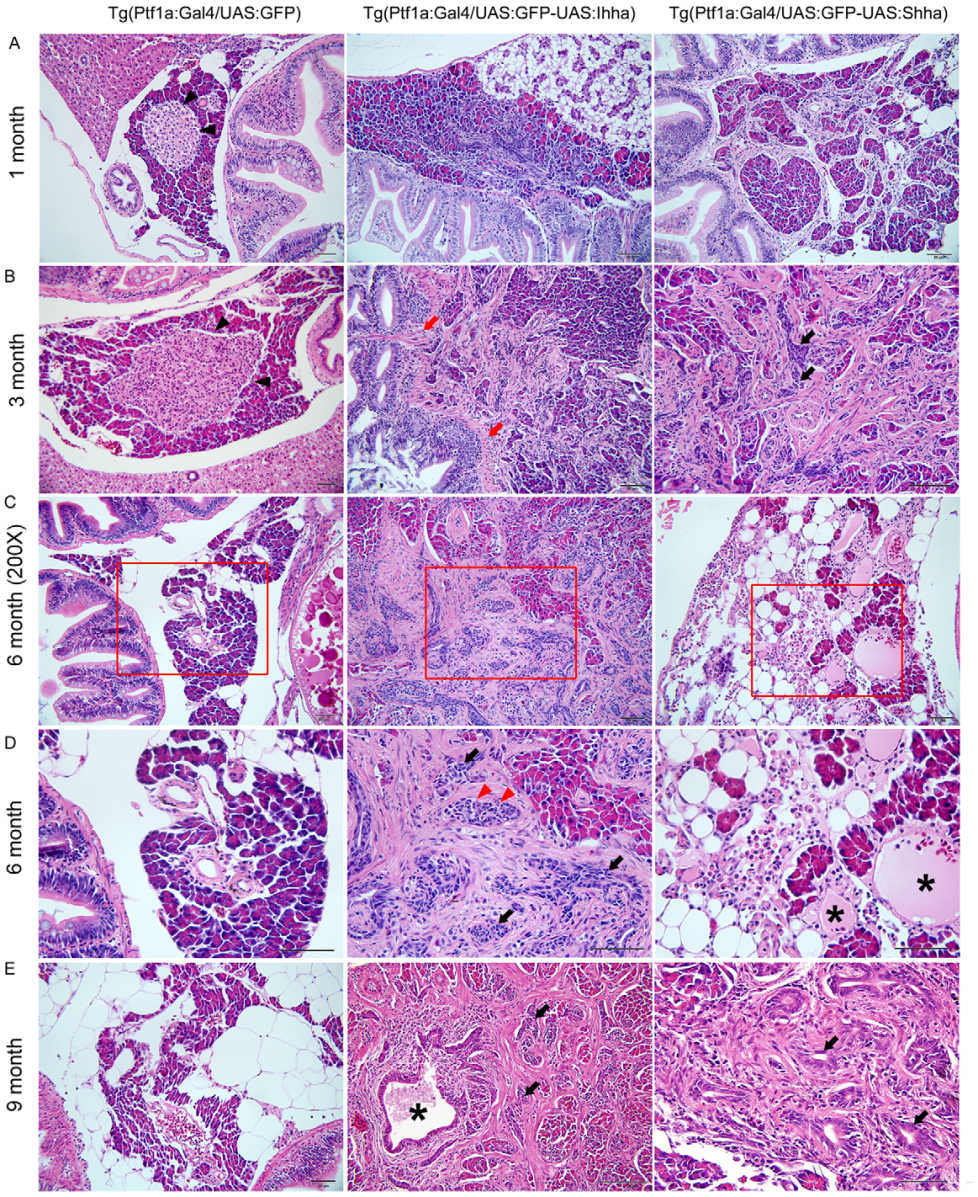
Histopathologic findings showing progressive pancreatic fibrosis. (**A**) Progressive pancreatic fibrosis starts at as early as 1-month old in Hh-expressing transgenic zebrafish. In non-fibrotic area, Individual morphology of the pancreatic acini and acinar cells is not unusual. (**B**) A principal islet is seen in control, which is well-circumscribed by acinar cells (black arrowheads). In Hh-secreting lines, accumulation of fibrosis results in the destruction of the morphologic architecture, which is prominent even at 3 months. Fibrotic bands are contiguous from the bowel wall forming adhesion between the bowel and the pancreas (red arrows), suggesting recruitment of myofibroblasts from the muscle layer of the bowel. Along with fibrosis, an increasing number of ductular structure appears within fibrotic area at 3 months of age (black arrows). (**C, D**) The pancreas at 6-months old. (D) An enlarged view of the red box in (C). Contrary to the islet of control in B, some islets of the Hh-expressing pancreas are completely encircled by fibrosis (red arrowheads), which is typical finding in chronic pancreatitis of human. The number of ductular structure further increased (black arrows). Occasionally, acute pancreatitis-like changes are noted, showing the infiltration of inflammatory cells and cystic space filled with mucinous material (asterisks). (**E**) The exocrine pancreas of 9 month-old zebrafish shows more accumulation of fibrosis and ductular structures (black arrows). At center image, a large pancreatic duct (asterisk) is seen, being surrounded by fibrosis and ductular structures. If not specified, microscopic images are 400×.Bars, 50 µm.

All Hh-expressing zebrafish from independent lines revealed a varying degree of pancreatic fibrosis and the desmoplasia was accumulated as the zebrafish aged (Fig. 3). Among the 3 groups of independent lines from each construct, we selected single representative line per group which revealed consistent and robust expression of transgenes. Both Ihh and Shh induced pancreatic fibrosis undistinguishable by histology alone. It is notable that Shh induced phenotypically more severe pancreatic fibrosis than Ihh at the given time points. The pancreatic fibrosis was progressive and manifested at as early as the age of one month (Fig. 3A). Fibrotic bands segregated and compartmentalized the exocrine glands, which resulted in the marked destruction of acinar structures at three months (Fig. 3B). Though typical lesions with fibrosis did not involve infiltration of inflammatory cells, transgenic zebrafish occasionally revealed inflammatory lesions similar to acute pancreatitis in humans, demonstrating infiltration of inflammatory cells, fluid collection, and necrosis (Fig. 3C,D). These findings, however, were unusual and appeared in less than 10% of the Hh-expressing zebrafish pancreas; therefore, it appeared to be caused by ductal obstruction resulting from fibrosis.

**Figure 4:**
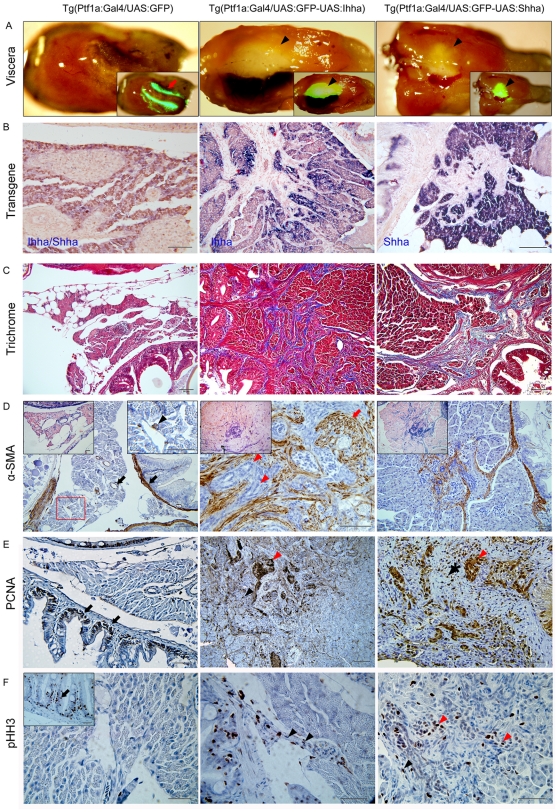
Hh-induced pancreatic fibrosis and proliferation of myofibroblasts. (**A**) Dissected whole viscera from 4 month-old zebrafish showing transgene (GFP) expression. Ventral views. Left, anterior. The pancreas of control appears as a thread-like structure between the bowel and visceral organs (arrow). In Hh ligand-expressing pancreas, prominent fibrosis around the principal islets forms whitish plaque-like lesions showing robust GFP expression (arrowheads). Inlets are merged in bright and fluorescence images. (**B**) ISH for transgene expression. The control pancreas reveals negligible expression of either Ihha or Shha. In the Hh-expressing pancreas, transgene expression is strictly restricted to acinar cells with nil expression at myofibroblasts or ductular cells. (**C**) Trichrome stains showing fibrotic bands. (**D**) IHC for α-SMA. Muscle layers of the bowel and large pancreatic ductal wall are reactive to α-SMA in control (black arrows). Infreqeuently, α-SMA-positive cells are noted (black arrowhead) in the parenchyme of control pancreas suggesting presence of stellate cells. Infilitrating myofibroblasts are invariably reactive to α-SMA while proliferating ductular cell are not (red arrowheads). Note the thickened and α-SMA-reactive intrapancreatic duct wall (red arrow). Left inlets (200×) are ISH images. Right inlet is an enlarged view of the box. (**E, F**) IHC for PCNA and pHH3. Within the fibrotic area, both ductular cells (red arrowheads) and myofibroblasts (black arrowheads) are frequently reactive to both PCNA and pHH3, suggesting enhanced proliferation. Intestinal crypt cells are also frequently reactive to both PCNA and pHH3 (black arrows) and used as internal control. If not specified, microscopic images are 400×. Bars, 50 µm.

The fibrotic changes were typically observed in the pancreas between the liver and gut, where the exocrine pancreas surrounded the principal islet. Interestingly, this corresponded to the area where the transgene expression was most robust. A prominent fibrotic area revealed a discernable whitish plaque in the entire dissected viscera and corresponded with the spot showing strong GFP expression (Fig. 4A). On ISH for Hh molecules which are co-expressed with GFP, transgene expression was strictly restricted to pancreatic acinar cells (Fig. 4B) which express a transcription factor Ptf1a over a lifetime. In the non-fibrotic area, however, the acinar and cellular morphology were well-preserved, suggesting acinar destruction was secondary to the accumulation of fibrotic change.

Proliferating myofibroblasts were invariably positive for α-SMA (Fig. 4D). The majority of the activated myofibroblasts seemed to come from the gut wall as the fibrotic strands were outstretching from the gut wall, forming an adhesion between the bowel and pancreas (Fig. 3B). α-SMA's reactivity was also noted in the muscle layer of the gut and pancreatic duct in control. Fibrotic bands found to be positive for α-SMA stain, also formed a contiguous strand from the gut wall (Fig. 4D), suggesting recruitment and activation of myofibroblasts from the muscle layers of the gut. Myofibroblasts in the pancreatic ductal wall were also activated and proliferated as the muscle layers thickened and expressed α-SMA (Fig. 4D). Occasionally, α-SMA-reactive cells were observed within the control pancreas, suggesting the presence of putative pancreatic stellate cells in the zebrafish pancreas (Fig. 4D). The source of proliferating myofibroblasts along with the preferential change in fibrosis around the principal islet suggested that Ihha or Shha recruited and activated any myofibroblasts in the vicinity of the pancreas where secreted Hh ligands could reach and mediate any effect.

Interestingly, proliferation of ductular structures was also noted at the age of three months, showing dense fibrotic bands intermingled with ductules (Fig. 3B). Along with the progression of fibrosis, the ductular structures had also accumulated within the fibrotic area. To see whether these ductular structures were formed by proliferation or by mere entrapment of existing ductules, IHC for PCNA and pHH3 was performed. The majority of ductular cells were strong-reactive to PCNA and many of them also expressed pHH3 (Fig. 4E, F), suggesting that the ductular structures were formed by enhanced proliferation.

The Hh signaling has been considered as a mediator of gastrointestinal tumorigenesis for many years, and Pdx1-Shh mice have shown metaplastic change and PanIn-like lesions [21]. However, the abnormal over-expression of Hh molecules did not cause pancreatic tumors in this study. We followed those Hh-expressing transgenic zebrafish for more than a year without finding any evidence of tumor foci or precancerous lesions.

### Differential genes involved in Hedgehog signaling and fibrosis

**Figure 5:**
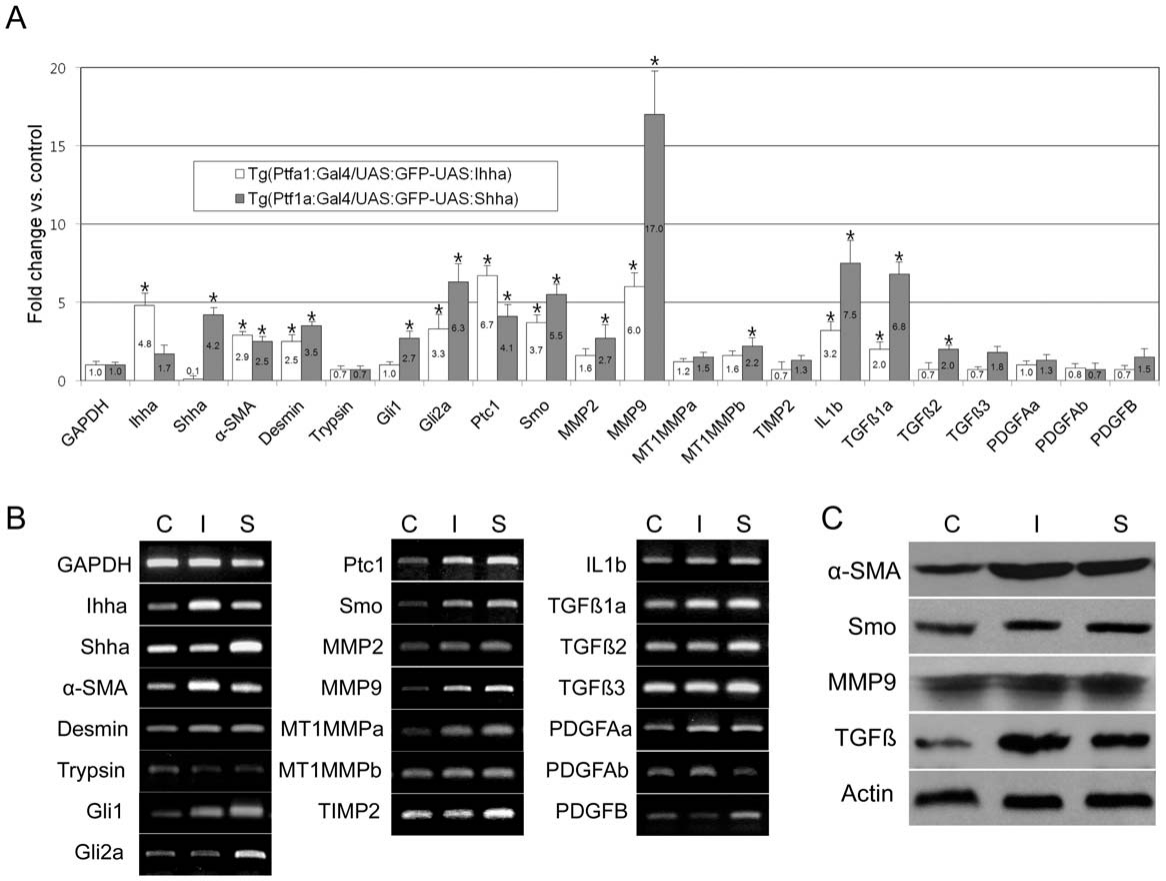
RT-PCR and Western blot. Pancreas from 3–4 month-old zebrafish was dissected under a fluorescence microscope. C, Tg(Ptf1a-Gal4/UAS:GFP); I, Tg(Ptf1a-Gal4/UAS:GFP-UAS:Ihha); S, Tg(Ptf1a-Gal4/UAS:GFP-UAS:Ihha). (**A**) Real-time RT-PCR showing differential expression of the components of the Hh pathway and fibrosis by Hh over-expression. Note the prominent up-regulation of MMP9 and TGFß1a. (**B**) Electrophoretic images of RT-PCR products recapitulate real-time PCR data. (**C**) A western blot hybridization using available antibodies which are reactive to zebrafish antigens also recapitulates RT-PCR findings. α-SMA, 42 kD; Smo, 85 kD; MMP9, 75 kD; TGFß, 45 kD; ß-actin, 45 kD. * P<0.05 versus control.

In order to identify differentially expressed genes, GFP-expressing pancreases were dissected under a fluorescence microscope and pancreas samples were extracted from 4–5 of each transgenic zebrafish, which were processed for RT-PCR. Among the Hh components, real-time RT-PCR revealed up-regulation in most of the downstream components including Ptc1, Smo, Gli1, and Gli2a as well as transgenes compared to the control, which suggested the presence of cells with active Hh signaling (Fig. 5A,B). The signaling pathways relevant to fibrosis comprise a long list of genes and gene families. We selected an exemplary list of genes that might have been modulated by aberrant expression of Hh ligands. Among the tested genes, RT-PCR revealed marked up-regulation of TGFß1aand MMP9, and mild to modest up-regulation of others, including membrane type 1 matrix metalloproteinase b (MT1MMPb), MMP2, interleukin1b (IL1b), TGFß2, and platelet derived growth factor Aa (PDGFAa) (Fig. 5A,B). A western blot hybridization was carried out using pooled samples from 4 month-old zebrafish with antibodies reactive to zebrafish antigen, which also recapitulated RT-PCR findings (Fig. 5C).

### Paracrine activation of responsive cells by Hedgehog ligands

**Figure 6:**
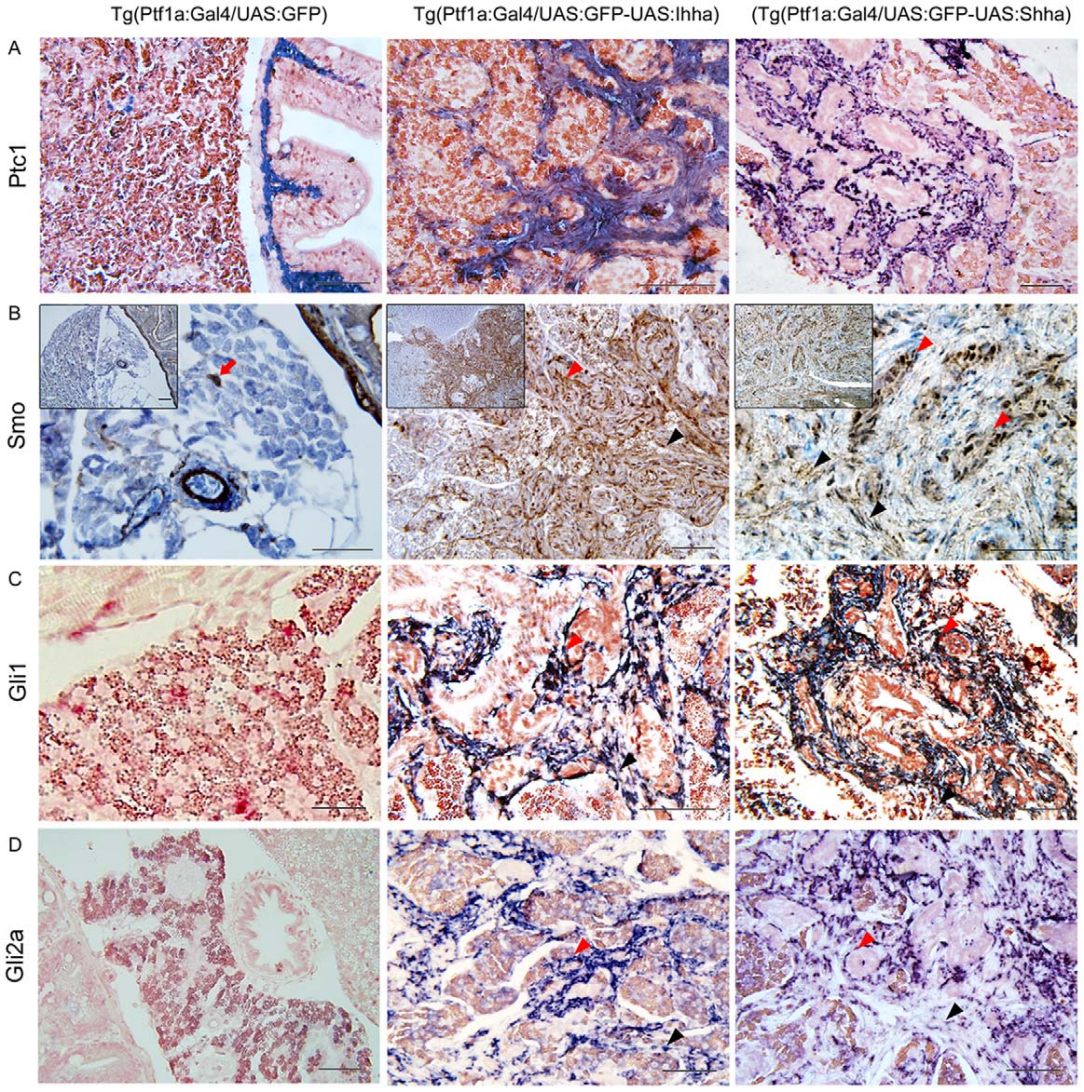
Expression of the downstream components of Hh signaling at 6 month-old zebrafish pancreas. (**A**) ISH for Ptc1. In control Ptc1 is expressed in the muscle layer of bowel and pancreatic duct. In the Hh-expressing pancreas, both proliferating myofibroblasts and ductular cells express Ptc1. (**B**) IHC for Smo reveals strong expression in a wide area of fibrosis. Both myofibroblasts and ductular cells are reactive to Smo. Likely to α-SMA immunostaining, Smo-reactive cells (red arrow) are occasionally noted in the parenchyma of the control pancreas. Inlets are 200× images. (**C, D**) ISH for Gli1 and Gli2a. Whereas, the control pancreas reveals a negligible degree of Gli1 and Gli2a expression, activated myofibroblasts and ductular cells express Gli1 and Gli2a. (**B–D**)Black arrowheads, myofibroblasts; Red arrowheads, ductular cells. If not specified, microscopic images are 400×. Bars, 50 µm.

Histologic expression of the Hh signaling components was assessed by either IHC or ISH, depending on the availability of an antibody that was cross-reactive to zebrafish antigen. Though Ptc1 theoretically counteracts the activation of Smo, the Hh ligand needs Ptc1 receptor to bind and initiate Hh signaling [25]. Ptc1 expression was restricted to proliferating myofibroblasts and ductular cells (Fig. 6A). The expression of Smo assessed by IHC was virtually identical to the Ptc1 expression (Fig. 6B). In control zebrafish, muscle layers of the bowel and pancreatic ducts also expressed both Ptc1 and Smo (Fig. 6A, B), suggesting paracrine activation of these Ptc1/Smo-positive cells by secreted Hh molecules. Similarly to α-SMA, Smo-reactive cells were occasionally noted within the control pancreas (Fig. 6B), which seemed to be the counterparts of pancreatic stellate cells. To further verify Hh signaling activation in responsive cells, we evaluated the expression of Gli genes, the final mediator of Hh signaling by ISH. The expression of both Gli1 and Gli2a was again strictly restricted to myofibroblasts and ductular cells (Fig. 6C, D). Even though, there exists a non-canonical pathway leading to the Gli1 expression [26], the Gli2 expression represents actual activation of the canonical Hh pathway [27], [28]. None of the acinar cells were reactive to Gli1 or Gli2a. The expression of Hh components in both myofibroblasts and ductular cells suggest that these two cellular compartments are responsive to Hh ligands secreted from acinar cells, activated, and proliferated to form dense fibrotic area intermingled with ductular structures.

### Hedgehog ligands induce MMPs and TGFß1 in Hedgehog-responsive cells

**Figure 7:**
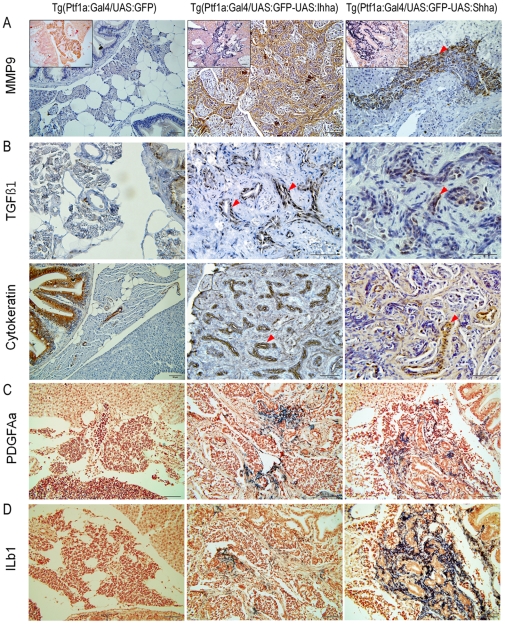
Expression of genes involved in fibrosis at 6 months. (**A**) IHC and ISH (inlets) showing MMP9 expression in proliferating myofibroblasts and ductular cells. (**B**) IHC for TGFß1. Contrary to MMP9, TGFß1 is expressed only in proliferating ductular cells which are also positive for cytokeratin. (**C, D**) ISH for PDGFAa and IL1b. Transcripts of both genes are detected in a small subset of proliferating myofibroblasts and ductular cells. (**A–C**, red arrowheads, ductular cells). Microscopic images are 400×. Bars, 50 µm.

MMPs function in the regulation of the extracellular matrix (ECM) organization by degrading ECM gives way to cellular migration. Thus, induction of MMPs is necessary for the progression of fibrosis. RT-PCR showed that induction of MMP9 was the most striking among the MMP genes evaluated in this study. While MMP2 was modest, MT1MMPs were mildly elevated. An immunostaining analysis revealed that both Hh-responsive myofibroblasts and ductular cells strongly expressed MMP9 with nil expression in acinar cells, which suggests that activated Hh signaling was responsible for induction of MMP9 (Fig. 7A).

TGFß family members also play important roles in fibrosis as well as tumorigenesis. Crosstalk between Hh and TGFß signaling has been found, and both genes are often co-expressed in epithelial compartments [13], [29]. Contrary to the MMP9, TGFß1 expression was strictly restricted only to proliferating ductular cells which were also reactive to the pan-cytokeratin antibody (Fig. 7B). This finding gives an important clue as to how active Hh signaling is involved in pancreatic tumorigenesis. Although, TGFß1 induction might have contributed to the aggravation of desmoplasia, it was not primarily responsible for fibrosis because ductular proliferation was not prominent until three months, when pancreatic fibrosis was already found. Unlike MMP9 and TGFß1, the expression of PDGFAa and IL1b was restricted to myofibroblasts (Fig. 7C, D).

### Phenotypic Reversal by Hedgehog Inhibitors

**Figure 8:**
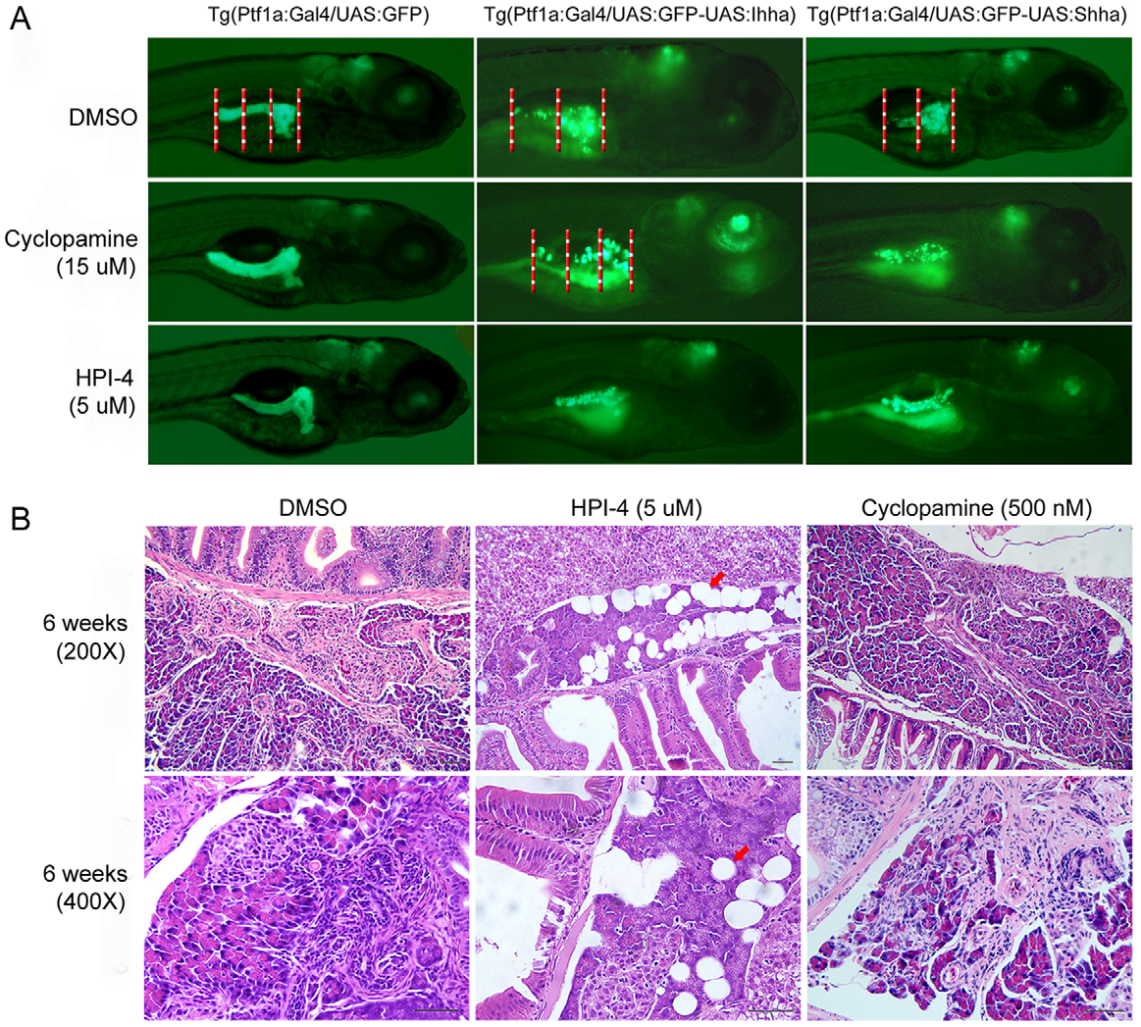
Phenotypic reversal by Hh inhibitors. (A) Reversal of pancreatic phenotypes in embryos. Embryos were treated with either HPI-4 or cyclopamine from 32 hpf until 5 dpf. Neither HPI-4 nor cyclopamine at the indicated concentrations impairs pancreatic development in controls. A well-formed pancreas in control produces a 1.5 to 2.0 times longer posterior pancreas compared to the head. Hh-expression induces a short and slender posterior pancreas showing the ratio between the body and head by approximately 1.0. By the criterion for reversal of the 1.5 times or longer posterior pancreas, the Hh-induced pancreatic phenotypes are effectively reversed by either HPI-4 or cyclopamine treatment. (**B**). Prevention of pancreatic fibrosis by a long-term treatment with Hh inhibitors. 12 day-old Ihha-expressing larvae were treated with Hh inhibitors for up to 6 weeks. In the HPI-4 treated group (12 out of 16 survived), there is no evidence of pancreatic fibrosis but a somewhat prominent fatty infiltration (red arrows). Contrary to HPI-4, cyclopamine failed to inhibit pancreatic fibrosis in the surviving 11 zebrafish out of 14. Bars, 50 µm.

The zebrafish model has been spotlighted for its feasibility in *in vivo* screening of candidate drugs due to a lower cost and a higher efficiency than with mouse models. To investigate the feasibility of phenotypic reversal by Hh inhibitors, Tg(Ptf1a-Gal4/UAS:GFP-UAS:Ihha) and control embryos were treated with the maximal tolerable dose (MTD) of either cyclopamine (15 uM, Smo inhibitor) or HPI-4 [30] (5 uM, ciliogenesis inhibitor working at downstream of Smo). Hh expression during embryonic periods induced pancreatic morphologic changes. Instead of a well-formed posterior pancreas in control, the Hh-secreting pancreas revealed a relatively prominent head with a short, slender, and tortuous posteriorly-growing pancreas. Whereas the length ratio of the posterior pancreas and head was between 1.5 and 2.0 in control at 5 dpf, it was roughly 1.0 in the Hh-expressing pancreas, which was used as criterion for reversibility. The pancreatic phenotypes were effectively reversed by either HPI-4 or cyclopamine treatment when evaluated by fluorescence imaging (Fig. 8A).

Next, groups of 12-day old Tg(Ptf1a-Gal4/UAS:GFP-UAS:Ihha) larvae were treated with Hh inhibitors for an extended period of up to 6 weeks. On histologic observation, the maximal tolerable dose (5 uM) of HPI-4effectively prevented pancreatic fibrosis but induced prominent fatty infiltration of the pancreas (Fig. 8B), which might need further investigation to have a further understanding of the underlying mechanism. However, contrary to HPI-4, cyclopamine failed to inhibit pancreatic fibrosis. This failure was possibly resulted from a low dose, due to dose-limiting toxicity (MTD: 500 nm in juvenile fish) or from a different mechanism itself as the HPI-4 directly disturbs ciliogenesis leading to the disruption of Gli1/Gli2 activity.

## Discussion

For the first time, current study presents a zebrafish model to study pancreatic fibrosis in which molecular events relevant to Hh-induced fibrosis can be explored. Zebrafish have recently been seen to simulate human disease in both molecular and histopathologic levels [18], [31]. In order to investigate the effect of Hh signaling in the pancreas, we have conducted an experiment in which zebrafish orthologs of Hh ligands are over-expressed in the Ptf1a domain. Along with a recent series of studies [10], [15], [20], [32], our result provides strong *in vivo* evidence that Hh signaling operates in a paracrine mode in the pancreas.

Among the three members of Hh ligands, Ihh and Shh expression is broader and strictly controlled in various organs, including the gastrointestinal system [5], [6], [33]; Dhh expression is largely restricted to the gonads during the development [3], [34]. Thus we chose subtypes of Ihh and Shh that are duplicated in zebrafish. Despite more prominent fibrosis by Shh expression, virtually identical phenotypes support functional redundancy between Ihh and Shh.

The canonical Hh pathway involves ligands, receptors, intracellular mediators, and transcription factors. In the present study, aberrant expression of Ihha and Shha molecules in the exocrine pancreas caused progressive fibrosis by paracrine action. This leads to the destruction of acinar structures which mimics desmoplasia occurring in human chronic pancreatitis and pancreatic cancer. Although the paracrine action of Hh signaling during embryonic development has been well-documented [14], [35], there have been debates on whether it works through a cell autonomous or non-autonomous mechanism, or both in pathologic conditions. *In vitro* studies have provided evidence of the autocrine activation of Hh signaling in keratinocytes, medulloblastoma, and renal cell carcinoma cells [17], [36], [37]. This is, however, not the case in the gastrointestinal tract and the pancreas, where Hh seems to work in an exclusively paracrine manner. Moreover, a similar mode of action has been demonstrated in fibrosis of the lungs and liver [11], [13], [38]. Other studies have also demonstrated that Hh molecules directly enhanced migration and proliferation of fibroblasts in those organs [10], [39]. Our study provides *in vivo* evidence that secreted Hh ligands cause pancreatic fibrosis by paracrine activation of responsive cells. The embryonic phenotypes in current models are not dramatic, they simply show morphologic changes of the exocrine pancreas. The development of the endocrine pancreas as well as other endodermal organs including the liver and the intestine were not affected by Hh over-expression. The exocrine markers such as trypsin, elastase, and CPA are properly and timely expressed in acinar cells. These findings strongly support the fact that Hh-expressing acinar cells are not influenced by this signaling but undergo proper differentiation. Although it is not clear whether the mode of action is dependent on cell type, the result suggests that a paracrine mechanism is highly involved in the pancreas.

Chronic pancreatitis and pancreatic cancer represent human diseases that are accompanied by progressive pancreatic fibrosis. In both conditions, Hh ligands are considerably over-expressed in metaplastic ductal and cancer cells and play central roles in desmoplasia (Fig. S2) [7], [8], [19]. Pancreatic stellate cells residing in the vicinity of the acini are the main source of proliferating fibroblasts in human disease. In the current models, the majority of the proliferating myofibroblasts in the pancreas seem to originate from the muscle layer of the bowel in the vicinity of the pancreas. Muscle layers of the large pancreatic ductal wall are a second source of proliferating myofibroblasts, as evidenced by thickened muscle layers which are immuno-reactive to α-SMA and Smo. The putative pancreatic stellate cells identified in the control pancreas by immuno-staining can be the third source of Hh-responsive cells. Therefore, it seems that Hh ligands indiscriminately recruit and activate myofibroblasts within the vicinity of Hh-secreting acinar cells. The pro-migratory effects of Hh signaling in multiple cell types have been well-documented, including neuronal and vascular endothelial cells as well as myofibroblasts [40], [41], [42], [43]. The activation of Hh signaling is concentration dependent, and secreted ligands are effective up to 300 um, which is the maximal distance they can reach by an unclear mechanism of molecular movement [44]. The close proximity between the pancreas and bowel in zebrafish allows secreted Hh molecules to reach and attract myofibroblasts from the gut wall. It is not clear if this phenomenon also occurs in the human pancreas, in which the distance between the pancreas and gut is much longer.

We also demonstrated that Hh-responsive myofibroblasts and ductular cells invariably express downstream components of Hh signaling. However, none of the acinar cells expressed these genes at either the mRNA or protein level. It is unclear as to how the Hh signaling exerts paracrine action in the pancreas, so it is crucial to determine responsiveness to secreted Hh molecules. In the current study, Hh-responsive myofibroblasts invariable expressed Ptc1 and Smo even in control, suggesting that expression of Ptc1 or Smo, or both determines Hh-responsiveness. Considering that Hh signaling is initiated by ligand-binding to the Ptc receptor, expression of the Ptc gene is mandatory for the initiation of Hh signaling. A recent observation has implicated that over-expression of Smo in pancreatic cancer-associated fibroblasts is a potential determinant for Hh-responsiveness [20]. It would be interesting to see whether forced co-expression of either Ptc1 or Smo, along with Hh ligands, might induce Hh responsiveness in acinar cells.

Our study suggests that the aberrant expression of Hh molecules does not induce tumors. We followed the transgenic zebrafish for more than one year without observing any evidence of tumor foci or PanIn-like lesions; as opposite to Pdx1-Shh mice that developed metaplastic duct and PanIn-like lesions with over-expressed Ptc1 and Smo [19]. The discrepancy may be attributed to the difference in the regulatory element driving Hh expression or to the biologic difference between teleosts and mammals. Otherwise, the metaplastic duct and PanIn-like lesions in Pdx1-Shh mice may actually be the counterparts of proliferating ductular structures found in our models.

We identified TGFß1 and MMPs as important mediators of Hh signaling. Recent observations have demonstrated that Hh signaling accelerates pancreatic tumorigenesis through tumor-stromal interaction by providing favorable conditions for tumor cells [21], [32]. The induction of TGFß1and MMPs expression in ductular cells gives an important hint as to how Hh signaling provides a favorable environment for tumor-stromal interaction during pancreatic tumorigenesis. Also, it provides theoretical background evidence that inhibition of Hh signaling is beneficial for the treatment of pancreatic cancer. In fact, recent observation has suggested that inhibition of Hh signaling can provide additional benefits to anti-tumor effects of conventional chemotherapy [45], [46]. A cross-sectional study has strictly demonstrated co-expression of TGFß1 and Hh molecules in epithelial compartments [13], and crosstalk between Shh and TGFß pathway has also been documented *in vivo* during embryonic development [47]. *In vitro* studies have shown that TGFß cooperates with canonical Hh signaling to activate Gli proteins and Hh target gene expression [48], [49], and exogenous Shh induces TGFß secretion in gastric cancer cells [50]. Therefore, the emergence of TGFß1-expressing ductular cells in current models harbors important implications. Though requisites for Hh-responsiveness need to be investigated, epithelial cells are indeed capable of responding to Hh ligands. Moreover, TGFß1 may be one of the targets that are induced by Gli-mediated transcriptional regulation, which may aggravate pre-existing conditions such as chronic pancreatitis and pancreatic cancer. The TGFß1 expression, however, is not primarily responsible for pancreatic fibrosis because fibrotic change was evident even at the first month when TGFß1-secreting ductular proliferation was not observed.

Similarly, MMPs play roles by remodeling the extracellular environment, which is an important step in the progression of fibrosis as well as tumorigenesis. In this study, Hh-responsive cells demonstrated the striking up-regulation of MMP9 with modest elevation of MMP2 and MT1-MMPs.This factor is consistent with *in vitro* observations that have demonstrated either exogenous Hh molecules or ectopic expression of Gli1 or Hh molecules induced MT1-MMP and MMP9 in cultured cells [51], [52]. MMPs induced by Hh signaling remodel the extracellular matrix and promote migration of activated Hh-responsive cells, which accelerates the fibrotic process. The *in vivo* environment enables exploration of epiphenomena manifested by the complex interaction of different types of cells. Thus, reflection of what really happens in the context of the physiologic and pathologic conditions is more than an *in vitro* study can provide.

While chronic pancreatitis accompanies the infiltration of inflammatory cells, the pancreatic pathology in these Hh-expressing zebrafish lacks an inflammatory reaction. Considering that the eventual pancreatic dysfunction in human chronic pancreatitis results from long-standing fibrotic change, acinar destruction and prevention of fibrosis is one of the main therapeutic targets. As a model, the zebrafish uniquely allows *in vivo* screening for small molecules, in which the effect of given drugs as well as toxicity can be simultaneously monitored under physiologic conditions. A visible short-term phenotype can facilitate high-throughput screening of candidate drugs. Though in this model, we could not thoroughly explain how the morphologies of a developing pancreas were changed by Hh expression, we could observe phenotypic reversal by treatment with Hh inhibitors. We also demonstrated that pancreatic fibrosis and destruction were effectively prevented by the treatment of HPI-4, a ciliogenesis inhibitor, but not with cyclopamine. Although failure by cyclopamine may be attributed to the dose limitation such as the high toxicity in the larval stage, this finding implies that targeting downstream of Smo may be beneficial for obtaining a therapeutic effect. Though the mechanism for differential sensitivity between embryos and larvae was unclear at the time, the acquisition of a toxicity profile in a physiologic context is an additional benefit of using the zebrafish as a model for drug screening. This study provides *in vivo* evidence that inhibition of Hh signaling is a viable option for the prevention of pancreatic fibrosis which has a detrimental effect on chronic pancreatitis and pancreatic cancer.

In conclusion, aberrant expression of either Ihha or Shha causes progressive pancreatic fibrosis through paracrine activation of Hh-responsive cells. We identified TGFß and MMPs as important genes induced by Hh signaling in responsive cells. These transgenic models will be a valuable platform in exploring the mechanism of fibrogenic pancreatic diseases caused by Hh signaling activation.

## Methods

### Ethics Statement

It was not necessary to obtain approval by the Laboratory Animal Committee at Yonsei University College of Medicine. The current committee does not request approval when non-mammalian models are used for experiments. This study, however, was strictly carried out to minimize suffering. All live images of embryos were taken under anesthesia using E3 media with 0.3 mg/mL tricaine. All adult zebrafish to be processed for experiments were euthanized by immersion in an ice-water bath.

### Transgenesis

Transgenic constructs were generated by modifying JD21-UAS:GFP-Kras, a kind gift from Steven D. Leach, which allows Tol2- to mediate transgenesis and is designed to co-express the transgene along with green fluorescence protein (GFP) which enabled real-time observation (Fig. 1A, Fig. S1). The cDNA for zebrafish Ihha (GenBank accession No. BC133983.1) was purchased from Openbiosystem Co., and zebrafish Shha (GenBank accession No. BC162395) was cloned using cDNA generated from three day-old wild-type embryos (AB line, ZIRC ZL1).While using polymerase with the proofreading function (Invitrogen), the GFP sequence including a polyA site was PCR amplified from pEGFP1 vector (Clontech) using F-GFP-Nco1/R-GFPpA-Xho1 primers. It was then digested and inserted into Nco1/Xho1 sites of JD21-UAS:GFP-Kras to generate JD21-UAS:GFPpA-Kras. Ihha and Shha were amplified with PCR usingF-Ihha-Mlu1/R-Ihha-Cla1 and F-Shha-Mlu1/R-Shha-Cla1 primers, respectively, then inserted into Mlu1/Cla1 sites of JD21-UAS:GFP-Kras, separately to generate JD21-UAS:Ihha and JD21-UAS:Shha. Each UAS:Ihha and UAS:Shha sequence was PCR amplified using F-UAS-Xho1/R-Ihha-Cla1 and F-UAS-Xho1/R-Shha-Cla1, respectively. It was then inserted into Xho1/Cla1 sites of JD21-UAS-GFPpAKras, separately, to generate the final transgene constructs JD21-UAS:GFP-UAS:Ihha and JD21-UAS:GFP-UAS:Shha.Schematic illustration for the generation of transgene construction is shown in Fig. S1. The control construct was generated by digesting JD21-UAS-GFPpA-Kras with Xhol1/Cla1, blunting, and then self-ligation. JD21-Ins-DsRed was generated for targeted expression of biomarker in pancreatic beta cells. Upstream a 1 kb sequence of the preproinsulin gene was PCR amplified from genomic DNA using F-Ins1 kb-Apa1/R-Ins1 kb-Nco1 primers and inserted into Apa1/Nco1 sites of JD21-UAS-GFPpA.Then, DsRed was PCR amplified from pDsRed-monomer-N1 (PT3795-5, Invitrogen Co.) using F-DsR-Nco1/R-DsR-Cla1 and inserted into Nco1/Cla1 site of JD21-Ins-GFPpA. All constructs were sequenced and verified using appropriate primers. Primers used for transgene constructs are listed in Table S1.

Each injection mixture was made by reconstituting Tol2-transposase mRNA (20 ng/ul) and a transgene construct (20 ng/ul) in Danieu's buffer mixed with 0.03% phenol red. Single-cell stage Tg(Ptf1a:Gal4) embryos were transferred to a molded agarose dish and 4 pL of injection mixture was introduced by yolk injection using a MMPI-2 micro injector. Approximately 50% of injected embryos survived. On day two, embryos showing GFP at the Ptf1a domain were selected using a fluorescence microscope, raised until adulthood, and out-crossed to generate F1 transgenic zebrafish. The utilization of Tol2-mediated transgenesis greatly enhanced the transgenic efficiency that 25–50% of F0 zebrafish from each construct gave rise to F1 offspring expressing transgenes. In each clutch of F1 embryos, approximately 10% showed transgene expression. Among the F1 progenies, embryos showing faithful expression were selected and raised to produce F2 progenies. All transgenes were transmitted into normal Mendelian ratios. Transgenic zebrafish were raised in a standardized aquaria system (Genomic-Design, Daejeon, Korea) according to standard protocols. Embryos to be processed for whole mount examination of GFP expression or ISH analyses were placed in 0.003% phenylthiourea at 24 hours post-fertilization (hpf) to inhibit pigmentation.

### Histology and Immunohistochemistry (IHC)

Histologic evaluation was performed in a subset of F2 transgenic zebrafish at 1,3,6,9, and 12-month(s). Hematoxylin and eosin (H&E) staining and IHC were performed according to the standard protocols. Primary antibodies used for immunohistochemistry were rabbit anti-α-smooth muscle actin (α-SMA) (Abcam ab15734, 1∶500), rabbit anti-Smoothened (Smo) (Abcam ab72130, 1∶200), rabbit anti-Gli1 (Upstate AB3444, 1∶500), rabbit anti-Gli2 (Abcam ab26056, 1∶300), mouse anti-Transforming growth factor ß1 (TGFß1) (R&D MAB1835, 1∶500), rabbit anti-matrix metalloproteinase 9 (MMP9) (Abcam ab38898, 1∶500), mouse anti-cytokeratin (CK) AE1/AE3 (Abcam ab961, 1∶500), mouse anti-proliferating cell nuclear antigen (PCNA) (Abcam ab29, 1∶1000), and rabbit anti-phosphohistone H3 (pHH3) (Cell Signaling 9701, 1∶200). Horse radish peroxidase (HRP)-conjugated secondary antibodies were utilized and colored using DAB solution. Slides were counterstained with hematoxylin, dehydrated, and mounted with Histomount (Zymed Co.).

### Western blot hybridization

A western blot hybridization was performed as previously described [53], using the exocrine pancreas dissected under a fluorescence microscope from 4 month-old zebrafish. The zebrafish pancreas does not form a single solid organ, but exists as thread-like structures being dispersed between visceral organs and embedded in fatty tissues. For each group, samples were collected from 20 to 30zebrafish and processed for protein extraction. Proteins were resolved by 10% SDSP-gels, blotted onto a nitrocellulose membrane, stained for 5 minutes with Ponceau S, blocked for 1 h in 5% milk in PBST, incubated over night at 4°C with a primary antibody in blocking buffer, washed 4 times with PBST, and incubated for 1 h with horseradish peroxidase-conjugated secondary antibody. Labeled proteins were detected by ECL reagents and Hyperfilm ECL (Amersham Biosciences).

### *In situ* hybridization (ISH) and whole mount immonofluorescence

ISH was performed either using 4% paraformaldehyde-fixed whole embryos or on 4-um sections of 4% paraformaldehyde-fixed, paraffin-embedded tissues as described previously [54]. To generate riboprobes, the corresponding coding sequences were PCR amplified from cDNA, TA cloned into pCRII vector (Invitrogen, CA, USA), and sequence-verified. Then, digoxigenin-labelled riboprobes were generated with the IVT kit (Roche Applied Science, Germany) using SP6 or T7 RNA polymerase depending on the orientation of the inserts. Primers used for TA cloning are listed in Table S2. Hybridized embryos or sections were bound with alkaline phosphatase-conjugated anti-Dig antibody, and colored using NBT/BCIP solution. Sections were counterstained with neutral red and mounted with Histomount.

Whole mount immunofluorescence was ISH was performed using 4% paraformaldehyde-fixed whole embryos essentially as described previously [54].Embryos were incubated overnight in10% goat serum with rabbit anti-CPA (Rockland, 100–4152), washed 3 times with PBST, and then incubated overnight in 10% goat serum with Cy3-conjugated anti-rabbit antibody (Jackson Labs). To identify individual acinar cells, photographs were obtained by using a Zeiss 700 confocal microscope with a 10× eye lens and a 20× objective lens.

### Imaging

Photographs were obtained using an Olympus BX51 for slide sections and an Olympus MVX10 for whole mount embryos. If not indicated, all section images were taken with a 10× eye lens and a 40× objective lens. If needed, zoom functions were used to obtain further magnified images.

### Semi-quantitative and quantitative reverse transcription-PCR (RT-PCR)

RT-PCR was performed using the exocrine pancreas dissected under a fluorescence microscope from three-month old zebrafish. For each group, samples were collected from five to six zebrafish and processed for RNA extraction. Real-time, quantitative RT-PCR was performed as previously described [18], using 7300 Real Time PCR System (Applied Biosystems, Foster city, CA) with the QuantiTectTMSYBRGreen PCR Kit (Qiagen, Valencia, CA). Samples were in triplicate, and all experiments were repeated three time using separately prepared samples. Statistical analysis was performed using SPSS 11 software. Statistical significance for quantitative RT-PCR was analyzed by the Mann-Whitney U test. Primer sequences are shown in Table S3.

### Treatment with Hedgehog inhibitors

To antagonize Hh signaling, either cyclopamine (Sigma-Aldrich Co., C4116) or Hh Primary Inhibitor-4 (HPI-4) (Sigma-Aldrich Co., H4541) was used [30]. For short-term phenotypic reversal, Tg(Ptf1a-Gal4/UAS:GFP-UAS:Ihha) embryos were treated in a petri dish from 32 hpf when Ptf1a expression first appeared in the primordial exocrine pancreas for five days with the maximal tolerable doses (MTDs) that would not impair embryonic development. MTDs were measured by treating embryos with a serial escalation of doses from 100 nM, which were 1 uM for HPI-4 and 15 uM for cyclopamine. Next, 12 day-old Tg(Ptf1a-Gal4/UAS:GFP-UAS:Ihha) larvae were treated in a 1L-breeding cage with Hh inhibitors for an extended period of up to six weeks. The MTDs (lethal in less than 25%) were measured again revealing 5 uM for HPI-4 and 500 nM for cyclopamine. Cage water was daily refreshed and inhibitors were newly added. At week six, juvenile zebrafish were processed for histologic evaluation.

## Supporting Information

Figure S1 **Schematic illustration for the generation of transgene constructs.**
(TIF)

Figure S2 **IHC for Hh ligands in human pancreas.** (**A, B**) Immunostaining for Ihh and Shh in a normal pancreas showing nil expression. (**C**) IHC for Ihh in chronic pancreatitis. Metaplastic ducts are strong positive for Ihh expression (arrows). (**D**) IHC for Shh in pancreatic cancer. Ductal cancer cells (arrow) and neighboring metaplastic ducts (arrowhead) are positive for Shh expression. Microscopic images are 400×.Bars, 50 µm.
(TIF)

Table S1 **Primers used for the generation of transgene constructs.** F-UAS-Seq was used for sequence verification of constructs. Underlined GCCACC sequence was inserted to satisfy Kozak sequence for proper transcription. Underlines, restriction enzyme sequences.
(DOCX)

Table S2 **Primers used for TA cloning to generate riboprobes.**
(DOCX)

Table S3 **Primers used for RT-PCR.**
(DOCX)
