# Supplementary figures and images for: Aberrant Hedgehog Ligands Induce Progressive Pancreatic Fibrosis by Paracrine Activation of Myofibroblasts and Ductular Cells in Transgenic Zebrafish
Source: PLoS One. 2011 Dec 2;6(12):e27941. doi: 10.1371/journal.pone.0027941 (PMC3229500; doi:10.1371/journal.pone.0027941)

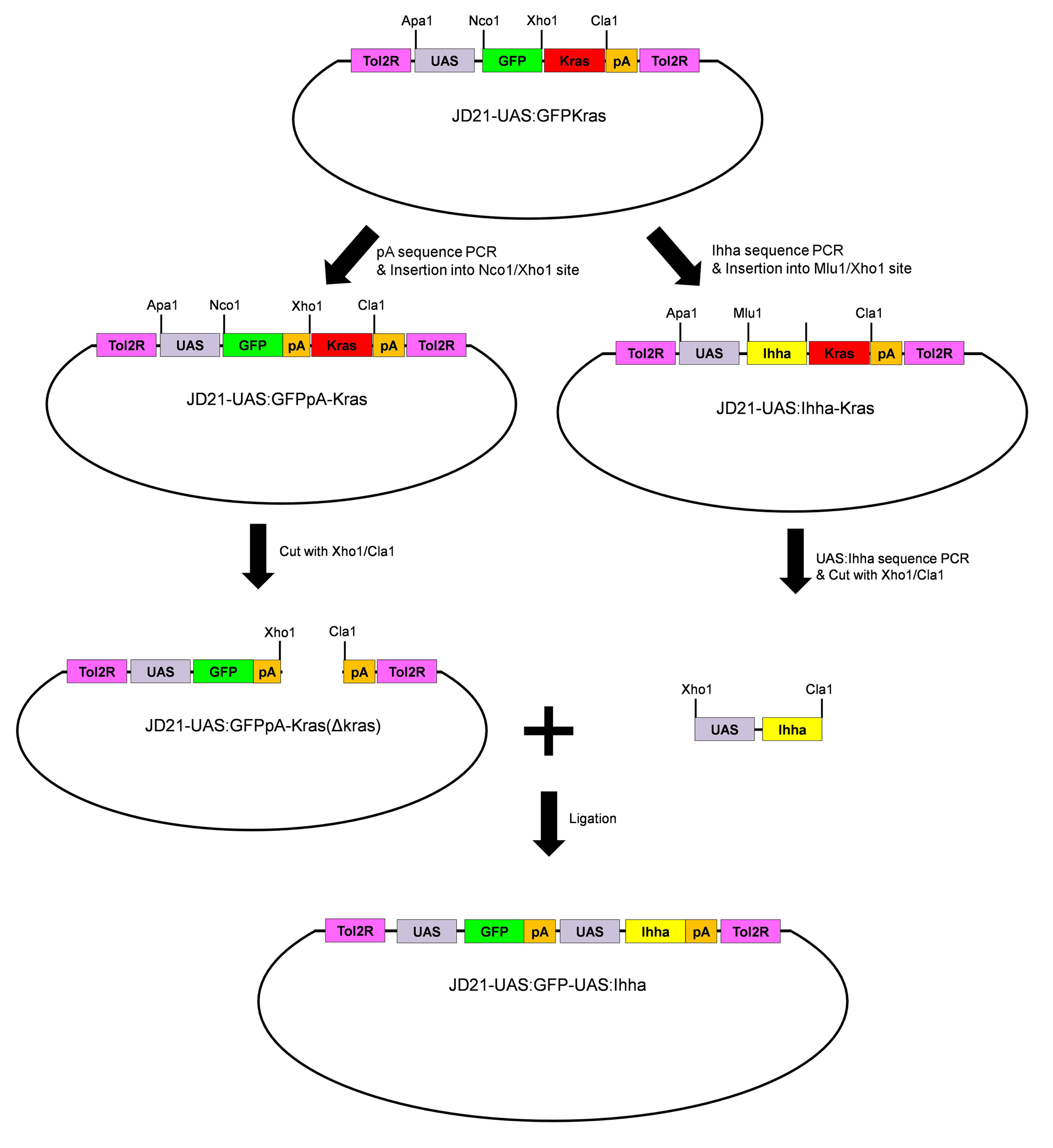

Supplement: Figure S1 — Schematic illustration for the generation of transgene constructs. (TIF) [file pone.0027941.s001.tif]

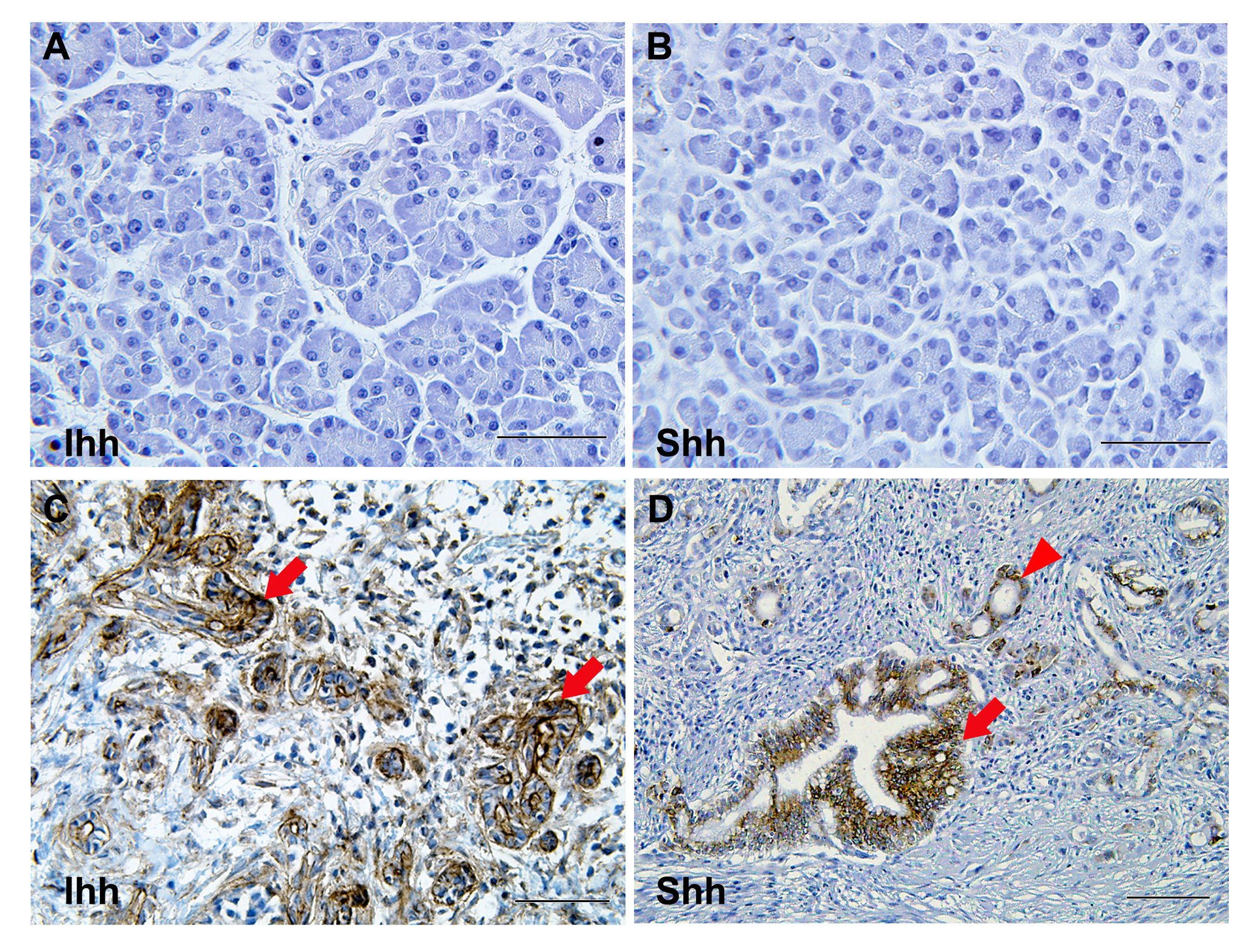

Supplement: Figure S2 — IHC for Hh ligands in human pancreas. (A, B) Immunostaining for Ihh and Shh in a normal pancreas showing nil expression. (C) IHC for Ihh in chronic pancreatitis. Metaplastic ducts are strong positive for Ihh expression (arrows). (D) IHC for Shh in pancreatic cancer. Ductal cancer cells (arrow) and neighboring metaplastic ducts (arrowhead) are positive for Shh expression. Microscopic images are 400×.Bars, 50 µm. (TIF) [file pone.0027941.s002.tif]
