# Supplementary material for: Aberrant Hedgehog Ligands Induce Progressive Pancreatic Fibrosis by Paracrine Activation of Myofibroblasts and Ductular Cells in Transgenic Zebrafish
Source: PLoS One. 2011 Dec 2;6(12):e27941. doi: 10.1371/journal.pone.0027941 (PMC3229500; doi:10.1371/journal.pone.0027941)
Supplement: Table S1 — Primers used for the generation of transgene constructs. F-UAS-Seq was used for sequence verification of constructs. Underlined GCCACC sequence was inserted to satisfy Kozak sequence for proper transcription. Underlines, restriction enzyme sequences. (DOCX) [file pone.0027941.s003.docx]

| Primers | Sequence |
| --- | --- |
| F-GFP-Nco1 | 5’-ATACCATGGTGAGCAAGGGCGAGGAG-3’ |
| R-GFP-Xho1 | 5’-ATACTCGAGATACATTGATGAGTTTGGAC-3’ |
| F-Ihha-Mlu1 | 5’-ATAACGCGTGCCACCATGCGTCTCCCCGTGGTGTT-3’ |
| R-Ihha-Cla1 | 5’-ACTAATCGATTCATCTATCATTGTCCATCA-3’ |
| F-Shha-Mlu1 | 5’-ATAACGCGTGCCACCATGCGGCTTTTGACGAGAGT-3’ |
| R-Shha-Cla1 | 5’-ACTAATCGATTCAGCTTGAGTTTACTGACA-3’ |
| F-Ins1kb-Apa1 | 5’-ACTAGGGCCCATTTAACTTCAGCCCACAGTCT-3’ |
| R-Ins1kb-Nco1 | 5’-CACACTGCCATGGTCACACT-3’ |
| F-DsR-Nco1 | 5’-ATACCATGGATGGACAACACCGAGGACGTC-3’ |
| R-DsR-Cla1 | 5’-ACTAATCGATCTACTGGGAGCCGGAGTGGCGGG-3’ |
| F-UAS-Xho1 | 5’-ATACTCGAGCTCTGCTAACCATGTTCATG-3’ |
| F-UAS-Seq | 5’-TCAGCCTCACTTTGAGCTCC-3’ |
