# Supplementary material for: Aberrant Hedgehog Ligands Induce Progressive Pancreatic Fibrosis by Paracrine Activation of Myofibroblasts and Ductular Cells in Transgenic Zebrafish
Source: PLoS One. 2011 Dec 2;6(12):e27941. doi: 10.1371/journal.pone.0027941 (PMC3229500; doi:10.1371/journal.pone.0027941)
Supplement: Table S2 — Primers used for TA cloning to generate riboprobes. (DOCX) [file pone.0027941.s004.docx]

| Genes | Sense (5’-3’) | Antisense (5’-3’) | Product length (bp) |
| --- | --- | --- | --- |
| Trypsin | ATGAAGGCTTTCATTCTTCTG | TCATGGTGTTTCTGATCCAG | 730 |
| Elastase | ATGTTCGCCCTCATCCTAGC | TTAGTTGTTCATCATGACCT | 813 |
| CPA5a | ATGAAGAGGCTGCTGGTGCT | TTAATAAGGGTTATTCTTGG | 1260 |
| Ptf1a | ATGGACACTGTGTTGGATCC | TTAGGAAATGAAATTAAAGGG | 798 |
| GATA6 | ATGTATCAGACCCTGGCCA | CCCACCAGTGTTGAAGAGT | 1007 |
| FoxA3 | ATGTTGAGCTCCGTGAAGAT | CGACTTGAGGTCCATCTTCT | 1176 |
| Ihha | ATGCGTCTCCCCGTGGTGTT | TCATCTATCATTGTCCATCA | 1242 |
| Shha | ATGCGGCTTTTGACGAGAGT | TCAGCTTGAGTTTACTGACA | 1257 |
| αSMA | GTGTGACGACGAAGAAAGCA | CTTCATCATACTCCTGCT | 1099 |
| Gli1 | AGTTCGTTTGCCACTGGAAG | ACGTTGCTCAAGCTGTTAAA | 1492 |
| Gli2a | GTTTCCCGAGTCCCAGACTG | CCAACTCCAGCAGAAGTACC | 1447 |
| Ptc1 | TGAAGCCTGAAACTAAGACTGT | TCAGGAGAAGGACTTTGCAA | 1363 |
| MMP9 | CTGCTCCATTGTTGGAAGCT | TTCTTTCCCACTCAGCTTGA | 1367 |
| IL1b | CATGCGGGCAATATGAAGTC | CTAGATGCGCACTTTATCCT | 818 |
| PDGFAa | CGCTGATCCACTTTCTCGTC | TCACCTTATATCTGCTGTGT | 578 |
