# Supplementary material for: Aberrant Hedgehog Ligands Induce Progressive Pancreatic Fibrosis by Paracrine Activation of Myofibroblasts and Ductular Cells in Transgenic Zebrafish
Source: PLoS One. 2011 Dec 2;6(12):e27941. doi: 10.1371/journal.pone.0027941 (PMC3229500; doi:10.1371/journal.pone.0027941)
Supplement: Table S3 — Primers used for RT-PCR. (DOCX) [file pone.0027941.s005.docx]

| Genes | Sense (5’-3’) | Antisense (5’-3’) | Product length (bp) |
| --- | --- | --- | --- |
| GAPDH | AAAGTCACCGCCATCAACGAC | CCTTAACCTCACCCTTGTACTT | 173 |
| Ihha | ATGCGTCTCCCCGTGGTGTT | TGGCTCCCAGTGTCTTCTCG | 175 |
| Shha | GCTTTTGACGAGAGTGCTGCT | TAAGGTCTTCTCCGCGACAT | 163 |
| α-SMA | GTGTGACGACGAAGAAAGCA | TTCTGCCCCATTCCTACCAT | 153 |
| Desmin | ACGAAATATTCAGCCTCCGC | GAGCTCTTGGTCACCTCGTA | 152 |
| Trypsin | GAAGGCTTTCATTCTTCTGGCTCTTT | GGTTGCTGATCAGAGAGCCA | 171 |
| Gli1 | ATGCCAGTGGATATGCAGCC | GGCCATGGAGGGATTATACA | 183 |
| Gli2a | ATGGAGACCACAAGTCCCAC | CTTCCTTCATGATGCCGCAT | 179 |
| Ptc1 | ATGGCCTCGGATCCCAGAGA | CCCACAGCTTTCCCCTTAGA | 167 |
| Smo | CAAGCGCCCCTGCTCCATTGTT | TGCGTGTACGGCAAAGGCGA | 186 |
| MMP2 | GTTGAAGGACACGCTGAAGAAA | GGGTGTGCCCTAAGATTCTG | 191 |
| MMP9 | ATGAGACTTGGAGTCCTGGC | TTAGCATTGGAGATGACCGC | 209 |
| MT1MMPa | ATGTTACCGAAACTGCAGACG | GATTTAGGAGAGCGAATCGC | 173 |
| MT1MMPb | ATGATCTGGAGCGGGTTTAC | CAGGCCGTAGAATCTCTGCA | 201 |
| TIMP2 | TGAAGAGCGTCAGGAGCTGTA | GCTTGATCGGGTTCCCATAA | 197 |
| IL1b | CATGCGGGCAATATGAAGTC | CATTTTGTGCTGCGAAGTCC | 170 |
| TGFß1a | GTTGGTTTGCTTGGTGCTGA | ATCTTCTGTCCGTCGTCGTC | 186 |
| TGFß2 | TGAACTTGTACGTCTTGAGCC | GATCTCAGGAGGACTGCTCA | 167 |
| TGFß3 | AAAGGACTGCTGTTTGTTCTG | ATCCCTGGTGCTGTTGTAGA | 216 |
| PDGFAa | CGCTGATCCACTTTCTCGTC | CGTCCTCCAGCACTTCATTC | 171 |
| PDGFAb | ATGAGAACCTTATTCTGCTGC | ATGGTGCTTCTGCTTGACCT | 207 |
| PDGFB | GGACCCTCTTCCTCCATCTCT | GGCTTCTGGGAAGACGTTTG | 164 |
